# 7. RETINAL FUNCTIONS EXPRESSED IN RETINAL IMAGING, CONTRAST PROCESSING AND ELECTRORETINOGRAPHY MAY DECRYPT EARLY RISK MECHANISMS AND PATHOPHYSIOLOGY OF SCHIZOPHRENIA AND MOOD DISORDERS AND ACCELERATE TRANSLATION TO THE CLINIC

**DOI:** 10.1093/schbul/sby014.022

**Published:** 2018-04-01

**Authors:** Michel Maziade

**Affiliations:** Centre de recherche CIUSSS de la Capitale-Nationale

## Abstract

**Overall Abstract:** The rationale of this symposium is twofold. First, true understanding of the neurobiological and environmental causes of schizophrenia and mood disorders will require the investigation of the human neuronal tissue in function. As an external and accessible extension of the brain, the retina opens this avenue. Existing technologies such as retinal imaging, computerized psychophysical assessment, and electroretinography (ERG) have recently provided evidence that the microanatomy of the retina and strength of rod, cone, and bipolar cell responses to light stimuli can distinguish patients from healthy individuals (Adams & Nasrallah, Schizophr Res 2017; Silverstein & Rosen, Schizophr Res Cogn 2015; Bubl, Biol Psychiatry, 2010; Plos ONE, 2015, Meier, Am J Psychiatry 2014). Second, the search for indicators of brain dysfunction that are detectable both in adult patients and in children at genetic risk is the cornerstone of genetic high-risk research into the neurodevelopmental origins of serious mental disorders and prevention (Maziade, New Eng J Med 2017). In this regard, it has been shown that children at genetic risk show many of the retinal anomalies that adult patients display (Hébert et al., 2010, Biol Psychiatry). Studies of the retina can therefore contribute to clarifying illness pathophysiology and its developmental roots.

This symposium objectives are to: 1) present findings from retinal imaging, visual processing and electroretinography in patients with schizophrenia or mood disorders, and in young healthy offspring of affected parents; 2) to discuss the data in terms of their implications for understanding psychotic and mood disorders; and 3) to clarify the similarities and differences between retinal findings in psychotic and mood disorders and their early developmental trajectories.

Participating scientists: Professor Michel Maziade will be the chair, with Professor Steven Silverstein as the co-chair of the symposium. Professor Anne Giersh, INSERM Strasbourg, France, will act as the discussant.

Professor Emanuel Bubl, Saarland University, Germany, will present findings on the potential of ERG measured retinal background noise as neurobiological correlate for cognitive deficits in ADHD and schizophrenia.

Professor Maziade, Laval University, Canada, will present new ERG findings in young offspring of parents affected by schizophrenia or bipolar disorder and the implications for the illness developmental origin and later transition to illness.

Professor Madeline Meier, Arizona State University, USA, will present results showing phenotypic and genetic associations between schizophrenia and retinal vessel diameter. Findings suggest that individuals with schizophrenia are at increased risk of microvascular complications.

Professor Silverstein, Rutgers University, USA, will present new ERG findings in schizophrenia, and data on the relationships between ERG anomalies, symptoms, retinal structural abnormalities as measured with optical coherence tomography, and antipsychotic medication use.

Based on empirical data, the symposium will offer an integrated view as to: i) how non-invasive measurements of retinal structure and function show consistent anomalies in schizophrenia, bipolar disorder, major depression and ADHD; ii) how findings from children and adolescents at high genetic risk not only indicate a neurodevelopmental process, but also suggest that retinal anomalies in patients are not due to medication use or degenerative effects; iii) how ERG can be administered to adults and children at low cost in clinical studies; iv) how to integrate findings in the staging of the risk status of children at genetic risk.

